# Endocytosis of AtRGS1 Is Regulated by the Autophagy Pathway after D-Glucose Stimulation

**DOI:** 10.3389/fpls.2017.01229

**Published:** 2017-07-12

**Authors:** Quanquan Yan, Jingchun Wang, Zheng Qing Fu, Wenli Chen

**Affiliations:** ^1^Ministry of Education Key Laboratory of Laser Life Science and Institute of Laser Life Science, College of Biophotonics, South China Normal University Guangzhou, China; ^2^Department of Biological Sciences, University of South Carolina, Columbia SC, United States

**Keywords:** *Arabidopsis thaliana*, D-glucose, AtRGS1, endocytosis, autophagy

## Abstract

Sugar, as a signal molecule, has significant functions in signal transduction in which the seven-transmembrane regulator of G-protein signaling (RGS1) protein participates. D-Glucose causes endocytosis of the AtRGS1, leading to the physical uncoupling of AtRGS1 from AtGPA1 and thus a release of the GAP activity and concomitant sustained activation of G-protein signaling. Autophagy involves in massive degradation and recycling of cytoplasmic components to survive environmental stresses. The function of autophagy in AtRGS1 endocytosis during D-glucose stimulation has not been elucidated. In this study, we investigate the relationship between autophagy and AtRGS1 in response to D-glucose. Our findings demonstrated that AtRGS1 mediated the activation of autophagy by affecting the activities of the five functional groups of protein complexes and promoted the formation of autophagosomes under D-glucose application. When the autophagy pathway was interrupted, AtRGS1 recovery increased and endocytosis of ATRGS1 was inhibited, indicating that autophagy pathway plays an important role in regulating the endocytosis and recovery of AtRGS1 after D-glucose stimulation.

## Introduction

In addition to integrating multi-faceted internal and external cues to gain nutrient homeostasis to build and fuel cells, sugars have significant hormone-like functions as primary messengers during signal transduction (Rolland et al., 2001, 2002; Ramon et al., 2008; Kang et al., 2010). In plants, a G-protein-coupled pathway is involved in sugar signaling. The *Arabidopsis thaliana* genome encodes an *Arabidopsis thaliana* regulator of G-protein signaling 1 (AtRGS1) protein that has an N-terminal seven transmembrane (7TM) domain and a catalytic RGS box at its C-terminal domain (Chen et al., 2003; Johnston et al., 2007; Urano et al., 2012; Bradford et al., 2013; Urano and Jones, 2014). In *Arabidopsis thaliana*, the heterotrimeric G-protein complex consists of one canonical Gα subunit (GPA1), one Gβ subunit (AGB1), and three Gγ subunits (Huang et al., 2015). AtRGS1 interacts with AtGPA1 and stimulates the rate-limiting GTPase activity of AtGPA1 in a D-glucose-regulated manner (Jeffrey et al., 2008). Previous studies by Urano et al. (2012) indicate that D-glucose recruits a with-no-lysing kinase (AtWNK) to phosphorylate AtRGS1 at least two C-terminal serines and that the phosphorylation of AtRGS1 is sufficient to cause endocytosis of AtRGS1 (Fu et al., 2014). Huang lab’s data reveals that deletion of the C-terminus (AtRGS^1-413^) has no effect on AtRGS1 localization at the plasma membrane (Hu et al., 2013). In our study, we would like to investigate whether autophagy pathway is involved in AtRGS1-mediated sugar signaling pathway.

Autophagy (‘self-eating’) is a highly conserved mechanism among eukaryotes for degrading and recycling intracellular materials during survival to several environmental stresses (Fujioka et al., 2008; Mizushima et al., 2011; Wang P. et al., 2016). Despite the clear role of autophagy in the innate immune system of the plant during infection by necrotrophic pathogens, recent studies have demonstrated that autophagy plays an important role in degrading and recycling intracellular molecules to regulate catabolic processes (Bassham, 2007; Li and Vierstra, 2012; Gao et al., 2016). When autophagy is induced, double-membrane vesicles (autophagosome) generate and engulf the cytoplasmic components, then transport them to the lysosome (animals) or vacuole (yeast and plants) for degradation. The degradation products of these cellular materials are released into the cytoplasm for recycling (Yang and Bassham, 2015; Wang X.D. et al., 2016). Autophagy likely has essential functions as protective mechanisms that assist plants to survive unfavorable growth conditions. Several distinct autophagic types have been defined in many species and classified as microautophagy, macroautophagy, chaperone-mediated autophagy, and organelle-specific autophagy (He and Klionsky, 2009; Liu and Bassham, 2012; Chanoca et al., 2015). Macroautophagy (hereafter, referred to as autophagy), as the most extensively studied autophagic type, is generated at the certain stage of development or upon encountering environmental stresses.

Several studies point to the relationship between RGS and autophagy pathway in animals. The Gα-interacting protein (GAIP), a member of a novel RGS family, is known to interact with the heterotrimeric G protein Gα_i3,_ to regulate inhibitory regulative G-protein (Gi) signaling pathways (Ogier-Denis et al., 1997). In human intestinal cells, GAIP/RGS19 acts as a GTPase-activating protein and inactivates the Gi protein, stimulating the lysosomal-autophagic pathway (Garcia-Marcos et al., 2011). In HT-29 cells, amino acids can mediate autophagy by inhibiting extracellular signal-regulated kinase1/2 (ERK1/2)-dependent GAIP phosphorylation (Ogier-Denis et al., 2000). Both GAIP and the Gα_i3_ protein are part of a signaling pathway that controls lysosomal autophagic catabolism. Phosphorylation of GAIP depends on the activation of the Erk1/2 MAP kinases. Activated ERK also abolishes the autophagy-inhibitory effects of the heterotrimeric Gi protein isoform Gi3 (Pattingre et al., 2003; Law et al., 2016).

Whether the AtRGS1-mediated sugar signaling pathway is related to the autophagy pathway during D-glucose stimulation remains elusive. Here, we show that autophagy is essential for regulation of the AtRGS1-mediated sugar signaling pathway in response to D-glucose in *Arabidopsis thaliana*. Furthermore, autophagy could promote the endocytosis of AtRGS1 and is involved in the recovery of AtRGS1 under D-glucose treatment. These results provide deep insights to the mechanism of AtRGS1 endocytosis upon D-glucose stimulation.

## Materials and Methods

### Plant Materials

The plants used in all experiments were *Arabidopsis thaliana* ecotype Columbia 0 (Col-0). The transgenic seeds (GFP tagged ATG8a, GFP-ATG8a adriven by 35S promoter) were provided by Dr. Li Faqiang (Biochemistry, City University of New York, New York, NY, United States). The T-DNA knockout lines *atg2* (SALK_076727), *atg5* (SALK_020601) were obtained from the *Arabidopsis* Biological Resource Center (ABRC). The 35S::AtRGS1-YFP (35S::AtRGS1 was subcloned into pEarleyGate101) *Agrobacterium* and At*rgs1–2* (SALK_074376) mutant seeds were kindly provided by Dr. Alan Jones (University of North Carolina, Chapel Hill, NC, United States). The overexpression transgenic seeds of AtRGS1-YFP, *atg*5/AtRGS1-YFP and *atg2*/AtRGS1-YFP were created by Dr. Wenli Chen in the laboratory of Alan Jones. Homozygousities of all DNA-insertion were confirmed by PCR analysis of genomic DNA with the primer sets listed in Supplementary Table S1 and **Figures S1B,C,E**. The transgenic lines were screened on MS plates with Basta (10 μg⋅mL^-1^) (**Supplementary Figure S1A**). The specific primers listed in Supplementary Table S1 were used to examine the gene expression levels of AtRGS1 described in **Supplementary Figure S1D**.

### Growth Conditions and Treatment

Approximately, 100 seeds of *Arabidopsis thaliana* were sterilized by sequential treatments with 75% (v⋅v^-1^) ethanol (1 min) and 1% (v⋅v^-1^) NaClO (10 min), followed by washing with sterile distilled water six times, vernalizing in dark conditions for 3 days at 4°C for better germination, and sown in liquid Murashige and Skoog (MS, without sucrose) medium with 1% D-glucose (pH 5.8). Seedlings were incubated in a plant growth chamber under dim continuous light (25 μmol m^-2^ s^-1^) at 23°C, shaking at 100 r⋅min^-1^ for another 5 days (Normal conditions). To sugar starve seedlings, the seedlings were then transferred to 500 mL flasks with 100 mL liquid MS medium without D-glucose or any other sugar and allowed to grow on a shaker (100 r⋅min^-1^) in the dark for 2 h. Following sugar starvation, seedlings were removed from the sugar-free MS medium and incubated with liquid MS media containing 1% or 6% D-glucose for the indicated time periods (0, 0.5, and 2 h) on a shaker (100 r⋅min^-1^) (Jeffrey et al., 2008; Urano et al., 2012).

### Gene Expression Analysis

Seedlings (0.1 g) were collected and frozen in liquid nitrogen and stored at -80°C by use of the TRIzol reagent according to the manufacturer’s instructions. The total isolated RNA was treated with primeScript RT Master Mix (Takara) according to the manufacturer’s instructions to synthesize the cDNA (Mackey et al., 2003; Caplan et al., 2008). Then, cDNA synthesis was performed by adding 5× PrimeScript RT Master Mix (Takara, 1× final concentration), total RNA (0–500 ng) and RNAse-free dH_2_O to a final volume of 10 μl, incubating the samples at 37°C for 15 min, followed by incubating at 85°C for 5 s to terminate the reactions. We amplified different autophagy-related genes to quantify transcript levels by using gene-specific primers (Supplementary Table S1) in seedlings exposed to different treatments. Real-time PCR (qRT-PCR) was performed in Applied Biosystems 7500 with the following thermocycler program: 1 min of pre-incubation at 95°C followed by 35 cycles of 15 s at 94°C, 30 s at 55°C, and 35 s at 72°C. SYBR Green dye fluorescence was used at the end of the annealing phase. To confirm the presence of single products, a melting curve from 65 to 95°C was used. The level of relative expression was analyzed by using the 2^-ΔΔCt^ analysis method (Sun et al., 2012).

### Confocal Microscopy

Root cells of seedlings located approximately in the elongation region were imaged using a Zeiss LSM 710 META system (LCSM; Carl-Zeiss, Jena, Germany). Confocal microscopy with excitation at 488 nm (a multi-Ar ion laser) and emission at 505–550 nm was used to detect the GFP-ATG8a fusion protein. YFP signals were excited by a 514-nm argon laser and its emission was detected at 520–550 nm by a photomultiplier detector, and for obtaining image quantification, ImageJ plugins was used. The digital Images were captured with a 40× oil immersion objective and analyzed with Aim Image Browser Image Processing software (Carl Zeiss) (Ishida et al., 2008; Zhang and Chen, 2011; Sheng et al., 2012).

### Protein Isolation and Immunoblotting

Plant samples (0.4 g) were ground in liquid nitrogen, re-suspended in ice-cold protein extraction buffer (10 mM Tris-HCl pH 7.5, 150 mM NaCl, 1 mM EDTA, 0.2% Triton X-100, 0.5% Nonidet p-40, 1 mM PMSF, 3 mM DTT, 0.5 mM CaCl_2_, 1% ASB-14 [For AtRGS1-YFP protein] and inhibitors), and then incubated on ice for 20 min. Samples were centrifuged at 12000 ×*g* at 4°C for 20 min. The supernatants were transferred to new 1.5 ml eppendorf tubes. Protein concentrations were measured by the Bradford method after the total protein was diluted 100 times. Sample buffer (5×, 25 μl) with 20 mM DTT was added to 100 μl of extracted proteins. The extracted proteins were vortexed lightly and incubated in 90°C boiling water for 2–5 min. Total proteins were then separated by SDS-polyacrylamide gel electrophoresis (PAGE) and transferred onto polyvinylidene difluoride membranes as described in earlier studies (Wang X.D. et al., 2016). The resulting membranes were blocked in TBST buffer with 5% (w/v) skim milk and incubated with the primary antibodies, followed by the corresponding secondary antibodies (Lianke^®^, Hangzhou, China). TBST with 5% (w/v) skim milk was used to dilute antibodies for immunoblotting. Antibodies for GFP (AG279) were purchased from the Beyotime Institute of Biotechnology (Shanghai, China); Antibodies for YFP (632381) was obtained from Clontech; Antibodies against ATG7 (ab99001) was purchased from Abcam (Shanghai, China). The levels of plant-actin were analyzed as controls for different treatments by western analysis using plant-actin antibodies (E12-053, Enogene^®^ Biotech, New York, NY, United States) (Yue et al., 2012). All digital images were analyzed in Image J when necessary.

### Statistical Analysis

All results were repeated at least three times. Statistical analysis was performed with an ANOVA in SPSS software. Statistical significance was accepted at the level of ^∗^*P* < 0.05, ^∗∗^*P* < 0.01.

## Results

### D-Glucose Activates the Autophagosomes

AtRGS1 serves as a receptor for glucose which induces its endocytosis (Grigston et al., 2008; Urano et al., 2012). Autophagy is a regulated vacuolar degradation pathway (Bassham, 2007). ATG8a protein is used as an alternative marker of autophagy. The green punctate structures, which could be labeled by GFP-ATG8a, are usually considered to be autophagosomes and their intermediates. The ATG8a protein always adheres to the autophagic vacuoles in the process of autophagic transport (Contento et al., 2005; Yoshimoto et al., 2009). To assess how the autophagy-related signaling pathway was activated in signal transduction pathways mediated by D-glucose, we examined root cells of GFP-ATG8a transgenic seedlings to see if autophagosomes were induced by D-glucose in *Arabidopsis thaliana*. Therefore, before and after D-glucose treatment, the green punctate structures were examined using laser confocal scanning microscopy. Concanamycin A (CA) is an inhibitor of the vacuolar type H^+^-ATPase (V-ATPase) that has been used to inhibit degradation by reducing the activity of vacuoles to stabilize autophagic cargoes (Kim et al., 2013; Shin et al., 2014). Before observation, the starved seedlings were treated with CA.

**FIGURE 1:**
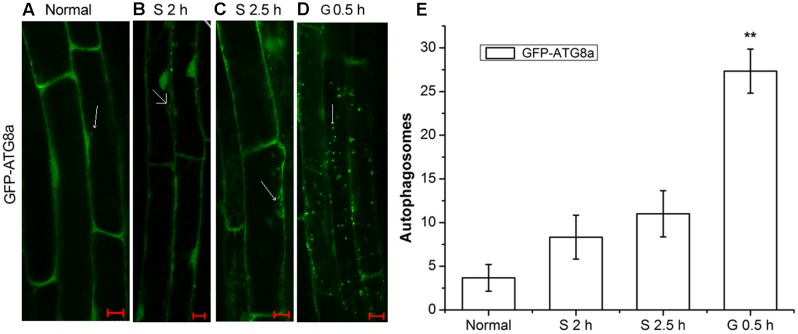
The observation of autophagosomes. Autophagosomes labeled by GFP-ATG8a in root cells of GFP-ATG8a plants with incubation in 1 μM CA. **(A)** Normal seedlings. **(B)** Starved seedlings for 2 h. **(C)** Starved seedlings for 2.5 h. **(D)** Starved seedlings for 2 h stimulated by 6% D-glucose for 0.5 h. **(E)** Quantification of the GFP-ATG8a-labeled autophagosomes per cell. Root cells at the indicated times was used to calculate the autophagic activity. The mean and SD values were calculated from roots of six seedlings per time point. Results in three parallel experiments were used for the quantification. The asterisks indicate significant differences from the starved seedlings treated with D-glucose for 0 h. Scale bars, 10 μm (^∗∗^*P* < 0.01).

As observed in **Figure 1**, in the root cells of GFP-ATG8a plants, a small number of autophagosomes were detected in starved seedlings for 2 h (**Figure 1B**) and 2.5 h (**Figure 1C**). Addition of D-glucose led to dramatic changes. There was a significant increase of autophagosomes. These results indicate that D-glucose could cause AtRGS1 endocytosis by acting as a signaling factor to activate the formation of autophagosomes.

### Induction of Autophagic Flux by D-Glucose

**FIGURE 2:**
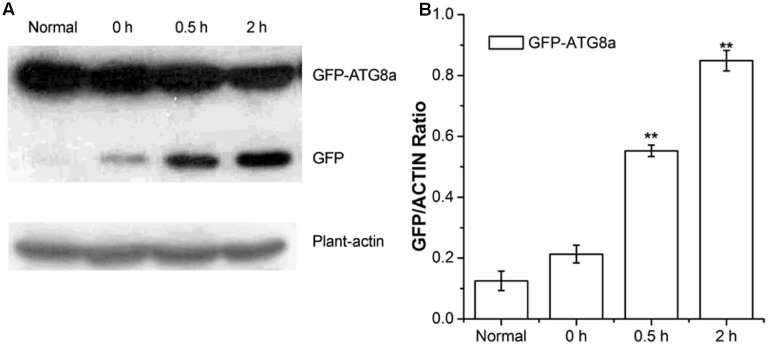
Analysis of autophagic flu in GFP-ATG8a. Five-day-old seedlings of GFP-ATG8a were starved by liquid MS medium without sugar following stimulated by 1% D-glucose for 0, 0.5, and 2 h. **(A)** Equal amounts of protein extracted from the seedlings were used to SD-PAGE, followed by western blotting with anti-GFP and anti-plant-actin antibodies. **(B)** Quantification of changes in free GFP normalized with the expression of plant-actin. Asterisks indicate significant differences from starved seedlings treated with 1% D-glucose for 0 h at ^∗^*P* < 0.05 or ^∗∗^*P* < 0.01. Error bar represent SD obtained from three independent replicates.

Autophagic flux was measured by western blotting using GFP antibodies in GFP-ATG8a transgenic *Arabidopsis* (Gao et al., 2016). During transport to the vacuolar lumen, GFP-ATG8, as a marker of autophagy, is often degraded to release free GFP, which was used to measure autophagic flux (Li et al., 2014). As shown in **Figure 2**, compared to the seedlings D-glucose treated for 0 h, seedlings treated for 0.5 and 2 h showed a higher level of GFP, in a time dependent manner (**Figure 2**). Our data indicate autophagic flux was induced by D-glucose in the plants of GFP-ATG8a, demonstrating that GFP-ATG8 was transported to the vacuolar lumen.

### Autophagy-Related Genes Expressions Rise in Wild-Type after D-Glucose Treatment

**FIGURE 3:**
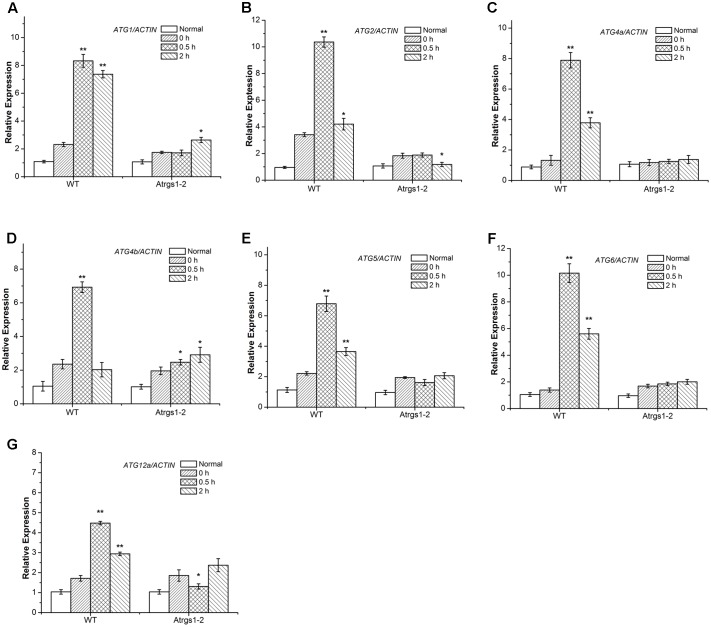
Expression of autophagy related genes in At*rgs1–2* and wild-type (WT) in response to D-glucose. Expression of *ATG1* **(A)**, *ATG2* **(B)**, *ATG5* **(C)**, *ATG4a* **(D)**, *ATG4b* **(E)**, *ATG6* **(F)**, *ATG12a* **(G)** in normal conditions and D-glucose treatment for 0, 0.5, and 2 h. Total RNA isolated from At*rgs1–2* and WT was subjected to qRT-PCR using gene-specific primers. Data represent mean and SD of at least three independent experiments. The asterisk indicates a significant difference from the starved seedlings treated with 1% D-glucose for 0 h (^∗^*P* < 0.05 or ^∗∗^*P* < 0.01).

Recently, several *Arabidopsis thaliana* autophagy-related (ATG) genes have been identified and their functions of them have been well-assessed (Liu and Bassham, 2012). Five functional groups, ATG1 composite enzyme complex, Beclin1-phosphatidylinositol 3-kinase (PI3K), ATG9 complex and two ubiquitination-like conjugation systems including ATG12-ATG5 and ATG8-PE, participate in the process of autophagosome formation (Hofius et al., 2011; Shpilka et al., 2015). To investigate the function of autophagy in the AtRGS1-mediated sugar signaling pathway in *Arabidopsis thaliana*, we studied the D-glucose-induced *ATG* gene expression in both At*rgs1*–*2* mutants and wild-type (WT) plants. qRT-PCR analyses showed that after D-glucose treatment, the transcript levels of all seven *ATG* genes were induced to a much higher level in WT plants than in At*rgs1–2* null plants, indicating that AtRGS1 is required for the expression of these seven *ATG* genes upon D-glucose stimulation (**Figure 3**). In addition, the induction of *ATG* was slower than At*rgs1–2* null plants than in WT plants. The expression level of *ATG1*, *ATG4b*, and *ATG12a* rose at 2 h in At*rgs1–2* (**Figures 3A,D,G**). However, in WT, the expression level of the *ATG* genes reduced when treated with D-glucose for 2 h, but still higher than the expression when treated with D-glucose for 0 h except *ATG4b* (**Figure 3D**). Therefore, we believe that AtRGS1 plays an essential role in D-glucose-induced expression of autophagy genes.

In the complicated process of the autophagosome formation, five functional groups of ATG proteins are needed to form autophagosomes at the certain stage (Thompson et al., 2005; Seay et al., 2006). The *ATG1* which encodes a protein kinase participates in the autophagy activation and autophagosome initiation (Kamada et al., 2010; Suttangkakul et al., 2011; Noda and Fujioka, 2015), and ATG6/ PI3K is essential for the nucleation of autophagic vacuoles (Patel and Dinesh-Kumar, 2008). We found a significant increase in the transcript levels of *ATG1* and *ATG6* in WT after 0.5 h D-glucose induction (**Figures 3A,F**), suggesting that autophagy was induced and autophagic vacuoles were nucleated. The ATG9 complex was sufficient for the membrane formation of autophagosomes. *ATG2* transcript level dramatically increased in response to treatment with D-glucose in WT (**Figure 3B**). The ATG12-ATG5 and ATG8-phosphatidylethanolamine (PE) conjugation systems are two ubiquitination-related conjugation system of autophagy, which are essential for the formation and closure of autophagic vesicles and the subsequent delivery to the vacuole (Thompson et al., 2005; Li et al., 2012; Ohsumi, 2014). As shown in **Figures 3C–E,G**, the increase of *ATG4a, ATG4b, ATG5*, and *ATG12a* expression after 0.5 h of stimulation suggested that the two ubiquitin-like protein-conjugating pathways were activated. Autophagy-related gene expression increased in WT, indicating that D-glucose had an important role in the formation of the five functional groups, and promoted the formation of autophagosomes.

### The Expression of ATG7 Protein Rises in Wild-Type upon D-Glucose Stimulation

**FIGURE 4:**
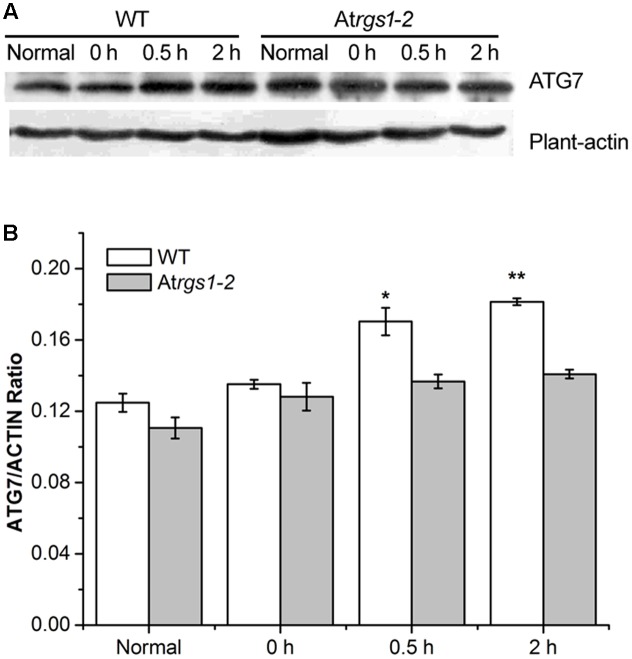
Immunoblots depicting the level of ATG7 protein in WT and At*rgs1–2*. Total proteins were extracted from treated seedlings at the indicated hours (normal seedlings, starved seedlings stimulated by 1% D-glucose for 0, 0.5, and 2 h). **(A)** Detection of ATG7 protein in WT and At*rgs1–2*. Equal amounts of protein were used to western blotting with anti-ATG7 antibodies, β-actin was subjected to the loading control. **(B)** Quantification of changes in ATG7 expression normalized with plant-actin. Images represented at least three independent experiments. Asterisks indicate significant differences from the starved seedlings treated with 1% D-glucose for 0 h at ^∗^*P* < 0.05 or ^∗∗^*P* < 0.01.

The E1-like ATG7-activating enzyme is required to form ATG8-phosphatidylethanolamine (PE) and ATG12-ATG5 conjugation systems, subsequently E2-conjugating enzymes, ATG3 and ATG10 combine ATG8 and ATG12 with PE and ATG5 (Wang P. et al., 2016). To elucidate the response of autophagy to D-glucose, we examined the autophagosome generation under D-glucose; ATG7 (76 kDa) protein levels were analyzed by western blot using an anti-ATG7 antibody. In WT, the expression of ATG7 protein significantly increased in response to D-glucose (**Figures 4A,B**). On the contrary, in At*rgs1–2* mutants, the expression of ATG7 only slight increased after D-glucose application (**Figures 4A,B**). The data obtained in the experiment suggested that AtRGS1 promoted the expression of ATG7 after D-glucose treatment.

### ATG2 and ATG5 Inhibit the Recovery of AtRGS1 in Response to D-Glucose

Autophagy is a precise and complex machinery used to degrade and recycle intracellular molecules in plants during response and survival to environmental stresses (Kulich et al., 2013; Bassham and Crespo, 2014). The disruption of the *ATG2* and *ATG5* gene blocks the formation of autophagosomes (Ishida et al., 2008; Phillips et al., 2008; Lee et al., 2014). AtRGS1, as a D-glucose receptor, mediates sugar signaling pathway by endosomes. To investigate the cellular function of autophagy during the AtRGS1 response to D-glucose, the levels of AtRGS1-YFP protein were assessed by the immunoblot analysis using an anti-YFP antibody in AtRGS1-YFP, *atg2*, and *atg*5 autophagy-deficient mutants.

**FIGURE 5:**
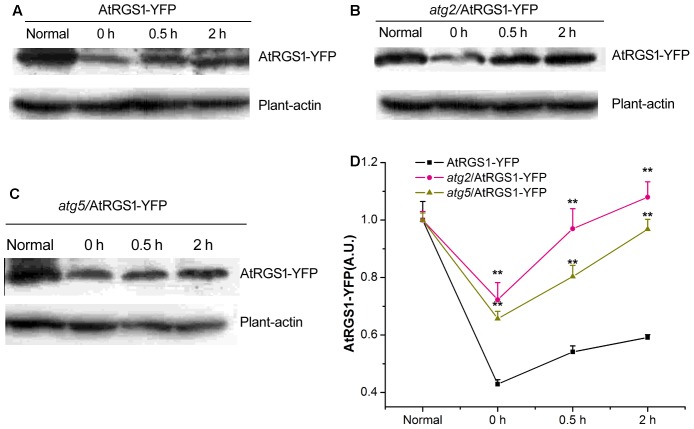
Detection of AtRGS1-YFP protein. The seedlings of AtRGS1-YFP **(A)**, *atg2*/AtRGS1-YFP **(B)**, *atg5*/AtRGS1-YFP **(C)** grown in liquid MS medium, then starved for 2 h, followed by 1% D-glucose application for 0, 0.5, and 2 h. Then the expression of AtRGS1-YFP protein were analyzed with anti-YFP antibodies. Plant-actin was used as the uniform protein loading. **(D)** Quantification of the level of AtRGS1-YFP protein following normalization to the expression of plant-actin in the three seedlings. Asterisks indicated significant differences from the seedlings of AtRGS1-YFP at each time point at ^∗^*P* < 0.05 or ^∗∗^*P* < 0.01.

Our data showed that AtRGS1-YFP protein expression levels were reduced after starvation, but, increased after D-glucose application in the plants of AtRGS1-YFP and the two autophagy-deficient mutants (**Figures 5A–D**). However, after 2 h starvation, AtRGS1-YFP protein in AtRGS1-YFP plants reduced more than that in the two mutants (**Figure 5D**). That means ATG2 and ATG5 promote the decrease of AtRGS1 protein under starvation treatment. After glucose application, AtRGS1-YFP protein recovery in AtRGS1-YFP plants was slower than that in *atg2* and *atg5* mutants (**Figure 5D**). These results demonstrated that ATG2 and ATG5 inhibit the AtRGS1 protein recovery after D-glucose treatment.

### ATG2 and ATG5 Promote the Endocytosis of AtRGS1 in Response to D-Glucose

**FIGURE 6:**
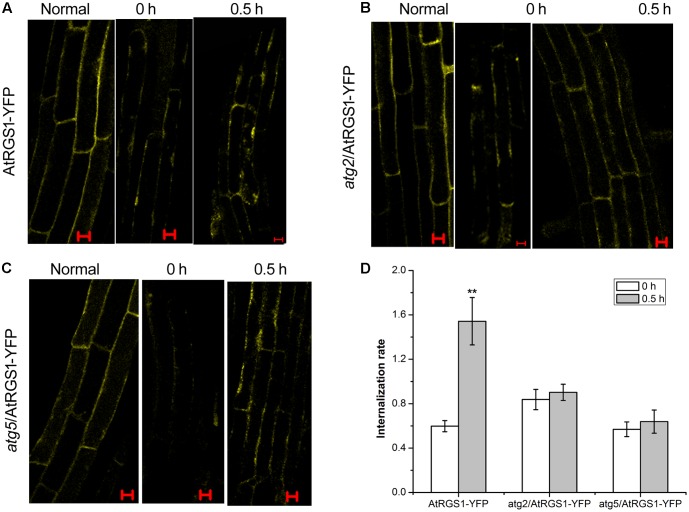
Confocal imaging of endocytosis of AtRGS1 in the plant of AtRGS1-YFP **(A)**, *atg2*/AtRGS1-YFP **(B)**, *atg5*/AtRGS1-YFP **(C)** (normal seedlings, starved seedlings stimulated by 6% D-glucose for 0 and 0.5 h). Scale bars represent 10 μm. **(D)** Quantification of AtRGS1-YFP internalization rate. Internalization rate was calculated by dividing the internalization of the starved seedlings treated with D-glucose for 0 or 0.5 h by the internalization of the normal seedlings. Experiments were performed three independent replicates with similar results. Asterisks, significant differences from the starved seedlings treated with D-glucose for 0 h, ^∗∗^*P* < 0.01.

It has been proven that D-glucose induces AtRGS1 endocytosis (Grigston et al., 2008). To determine whether autophagy functions to mediate endocytosis of AtRGS1 after D-glucose application, we analyzed the subcellular distribution of YFP fluorescence by using laser confocal scanning microscopy in AtRGS1-YFP, *atg2* and *atg*5 plants. In AtRGS1-YFP plants, endocytosis occurred after D-glucose application (**Figure 6A**). But, in *atg*2 and *atg*5 mutants, AtRGS1-YFP was mainly localized to the plasma membrane, and the internalization rate increased only slightly in response to D-glucose (**Figures 5B–D**). The results were consistent with the movement of AtRGS1-YFP in AtRGS1-YFP, *atg2* and *atg*5 plants (**Supplementary Figure S2**). These results revealed that under the stimulation of D-glucose, when the autophagy pathway was deficient in *atg2* and *atg*5 plants, the endocytosis of AtRGS1 was inhibited. AtRGS1 is mainly localized on the plasma membrane. Our data demonstrated that means ATG2 and ATG5 promote AtRGS1 endocytosis.

## Discussion

AtRGS1, which is proposed to be an extracellular receptor for D-glucose, accelerates the hydrolysis of AtGPA1 to negatively regulate G-protein signaling (Urano et al., 2012). Our study indicated that D-glucose could activate autophagy and that autophagy pathway did not function well in At*rgs1–2* mutants. The rapid recovery of AtRGS1 in *atg2* and *atg5* mutants suggested that the autophagy pathway might be involved in inhibition of the recovery of AtRGS1 in response to D-glucose. AtRGS1 endocytosis was inhibited and it mainly located on the plasma membrane in plants of *atg2* and *atg5*, indicating that ATG2 and ATG5 promote the endocytosis of AtRGS1. In this study we have demonstrated that autophagy is required for regulation of the AtRGS1-mediated sugar signaling pathway in response to D-glucose.

### The Autophagy Pathway Is Induced by D-Glucose

In plants, glucose as a metabolite or signaling molecule affects gene and protein expressions, and growth and developmental programs (Sheen, 2014; Huang et al., 2015). Autophagy is an intracellular degradation system conserved in eukaryotic cells that consists of the formation of double-membrane structures called autophagosomes that engulf and sequester the cytoplasmic components and then fuse them with the endosome/vacuole and finally break them down in the vacuole (Bassham, 2007; Wang P. et al., 2016).

Punctate-like structures labeled by GFP-ATG8a, which represent autophagosomes and their intermediates, appear in large numbers after treatment with D-glucose (**Figure 1**). Autophagic flux is often utilized to measure the degradation activity of autophagic substrates within the vesicular system (Mizushima et al., 2010; Loos et al., 2014). The increase of the accumulation of free GFP dependent on time implied that autophagic flux was induced by D-glucose in GFP-ATG8a plants (**Figure 2**), suggesting that autophagosomes were transported to the vacuole for breakdown. The assembly of five functional groups of ATG proteins is required for the formation of autophagy (Obara and Ohsumi, 2011). The increase of the expression autophagy-related gene expression in WT indicated that D-glucose promoted formation of autophagosomes (**Figure 3**). E1-like ATG7-activating enzyme is essential for the formation of ATG8-phosphatidylethanolamine (PE) and ATG12-ATG5 conjugation systems (Wang P. et al., 2016). After induction with D-glucose, the increased level of ATG7 protein in WT promotes the formation of two ubiquitination conjugation systems (**Figure 4**). Previous research has shown that the autophagic flux was inhibited in *atg7* mutants in *Arabidopsis thaliana* (Shin et al., 2014; Wang X.D. et al., 2016). In WT, the increased level of ATG7 protein induced by D-glucose will likely promote the autophagic flux (**Figure 4**). Taken together, these results indicate that D-glucose induces autophagy, which plays an important role in intracellular homeostasis.

### AtRGS1 Is Required for Autophagy Pathway Induced by D-Glucose

In *Arabidopsis thaliana*, three signaling pathways that are sensitive to glucose have been analyzed. (1) The hexokinase 1 (AtHXK1) pathway: AtHXK1 is recognized as glucose sensor involving in the metabolic and physiological processes by mediating gene transcription in response to glucose (Sheen, 2014). (2) The glycolysis-dependent SNF1-RELATED KINASE1/TARGET OF RAPAMYCIN (SnRK1/TOR) pathway: the glycolysis-dependent SnRK1/TOR pathway acts as cellular energy sensors regulated growth and development (Lastdrager et al., 2014). (3) The AtRGS1-dependent G-protein-coupled signaling pathway: G-protein-coupled pathways involving AtRGS1 are involved in sugar signaling (Grigston et al., 2008). Genetic evidence suggests that D-glucose causes AtRGS1 endocytosis or a sugar metabolite regulates AtRGS1 activity toward AtGPA1 (Urano et al., 2012).

Our results demonstrate that D-glucose induces autophagy pathway in WT *Arabidopsis* plants (**Figures 1–4**). However, in At*rgs1–2* mutants after D-glucose application, the analysis of qRT-PCR data indicate that the autophagy pathway did not function well (**Figure 3**), and the expression of ATG7 increase only slightly (**Figure 4**). The E1-like ATG7-activating enzyme is required to form the two ubiquitin-like protein-conjugating pathways (Wang P. et al., 2016), so we hypothesize that D-glucose induces the formation and activation of ATG8-phosphatidylethanolamine (PE) and ATG12-ATG5 conjugation systems, which are closely related to AtRGS1. A lot of evidence (**Figures 3, 4**) has been provided in support of the relationship between autophagy and AtRGS1 protein. Autophagy has been shown to be involved in AtRGS1-mediated sugar signaling pathway.

### ATG2 and ATG5 Regulate the Recovery and Endocytosis of AtRGS1 after D-Glucose Stimulation

Autophagy appears to be the main contributor to the maintenance of the equilibrium with different environmental stresses and the degradation and reuse of nutrients (Li et al., 2012; Liu and Bassham, 2012; Ohsumi, 2014; Wang P. et al., 2016). Endocytosis is a major route of entry for plasma membrane proteins or extracellular materials into the cell for recycling back to the plasma membrane, or degradation in the vacuole. Endocytosis plays an important role in the process of cellular responses to environmental stimuli and signaling transduction (Fan et al., 2015; Michaeli et al., 2016).

In mammalian cells, autophagosomes probably fuse with endosomes of GPCRs induced by agonists for degradation, or endosomes of GPCRs involve recycling via the endosome. In plants, the AtRGS1 interacted with some relative proteins stimulates the endocytosis of AtRGS1 and promotes downstream signaling pathway (Urano et al., 2012). AtWNK kinases recruited by D-glucose phosphorylate AtRGS1 to mediate endocytosis (Urano et al., 2012). The interactions between AtRGS1 and small GTPases molecules of RAB (Ras-like small GTP binding) and ARF (ADP-ribosylation factor) are hypothesized to regulate AtRGS1 to be endocytosed into the cytoplasm, then recycled back to the plasma membrane via recycling endosomes (Jaiswal et al., 2015).

In *Arabidopsis thaliana*, D-glucose induced the increase of the levels of AtRGS1-YFP protein expression in the plants of AtRGS1-YFP and *atg2* and *atg5* mutants (**Figures 5A–D**). After D-glucose application, AtRGS1-YFP proteins recovered more rapidly in *atg2* and *atg5* mutants than that in AtRGS1-YFP plants, indicating that ATG2 and ATG5 inhibited the AtRGS1 protein recovery after D-glucose treatment. Therefore, we conclude that autophagy pathway regulates AtRGS1 recovery (**Figure 5**).

However, in autophagy-deficient *atg2* and *atg5* mutants, the endocytosis of AtRGS1 was inhibited, indicating that ATG2 and ATG5 promote the endocytosis of AtRGS1 in response to D-glucose (??). Endocytosis of AtRGS1 physically uncouples the GTPase-accelerating activity of AtRGS1 from the G protein, which permits sustained activation (Urano et al., 2012). Perhaps inhibition of endocytosis of AtRGS1 leads to a physical coupling of AtRGS1 with AtGPA1 and concomitant inhibition of G-protein signaling.

In plants, endocytic and autophagic pathways interplay. Autophagosomes fuse with the endosome/vacuole, and degrade in the vacuole (Zhuang et al., 2015). So we hypothesize endocytosis of AtRGS1 and autophagic pathways interplay in *Arabidopsis thaliana*, and autophagic pathways have an effect on the recycling back to the plasma membrane, or degradation in the vacuole of AtRGS1.

Further study aims to determine whether AtRGS1-YFP co-localized with autophagic bodies after D-glucose stimulation. This study is essential for extending our knowledge of the relationship between AtRGS1 and autophagy.

## Author Contributions

QY and WC conceived and designed the experiments. QY and JW performed research. QY, ZF, and WC analyzed the data. QY and ZF wrote the manuscript.

## Conflict of Interest Statement

The authors declare that the research was conducted in the absence of any commercial or financial relationships that could be construed as a potential conflict of interest.

## Supplementary Material

FIGURE S1 **(A)** The transgenic lines (AtRGS1-YFP, *atg2*/AtRGS1-YFP and *atg5*/AtRGS1-YFP) and WT were screened on MS plates with Basta (10 μg/mL). DNA was respectively isolated from eight seedlings (1–8) of *atg2*/AtRGS1-YFP **(B)**, *atg5*/AtRGS1-YFP **(C),** and Atrgs1–2 **(E)**. WT acts as a control. Homozygous for DNA-insertion were confirmed by PCR of genomic DNA with the primers. The products were electrophoresed on a 1% gel for 30 min, then detected by using UV illumination. **(D)** qRT-PCR was performed to analyze transcription level of AtRGS1 in AtRGS1-YFP, *atg2*/AtRGS1-YFP and *atg5*/AtRGS1-YFP lines grown on MS plates for 7 days. Data represent mean and SD of at least three independent experiments. The asterisk indicates a significant difference from WT (^∗∗^*P* < 0.01).

FIGURE S2 Movement of AtRGS1-YFP in the plant of AtRGS1-YFP **(A)**, *atg2* **(B)**, *atg5* **(C)**. Root cells of seedlings located approximately in the elongation region were imaged using a Zeiss LSM 510 confocal laser scanning microscope (LCSM, LSM 510/ConfoCor 2, Carl-Zeiss, Jena, Germany). The normal seedlings were observation in response to D-glucose. Scale bars represent 10 μm. **(D)** Quantification of AtRGS1-YFP internalization rate. Internalization rate was calculated by dividing the internalization of the normal seedlings treated with D-glucose for 5, 10, or 15 min by the internalization of the normal seedlings treated with D-glucose for 0 min. Experiments were performed three independent replicates with similar results. Asterisks, significant differences from the starved seedlings treated with D-glucose for 0 min, ^∗^*P* < 0.05 or ^∗∗^*P* < 0.01.
