# Supplementary figures and images for: Endocytosis of AtRGS1 Is Regulated by the Autophagy Pathway after D-Glucose Stimulation
Source: Front Plant Sci. 2017 Jul 12;8:1229. doi: 10.3389/fpls.2017.01229 (PMC5506085; doi:10.3389/fpls.2017.01229)

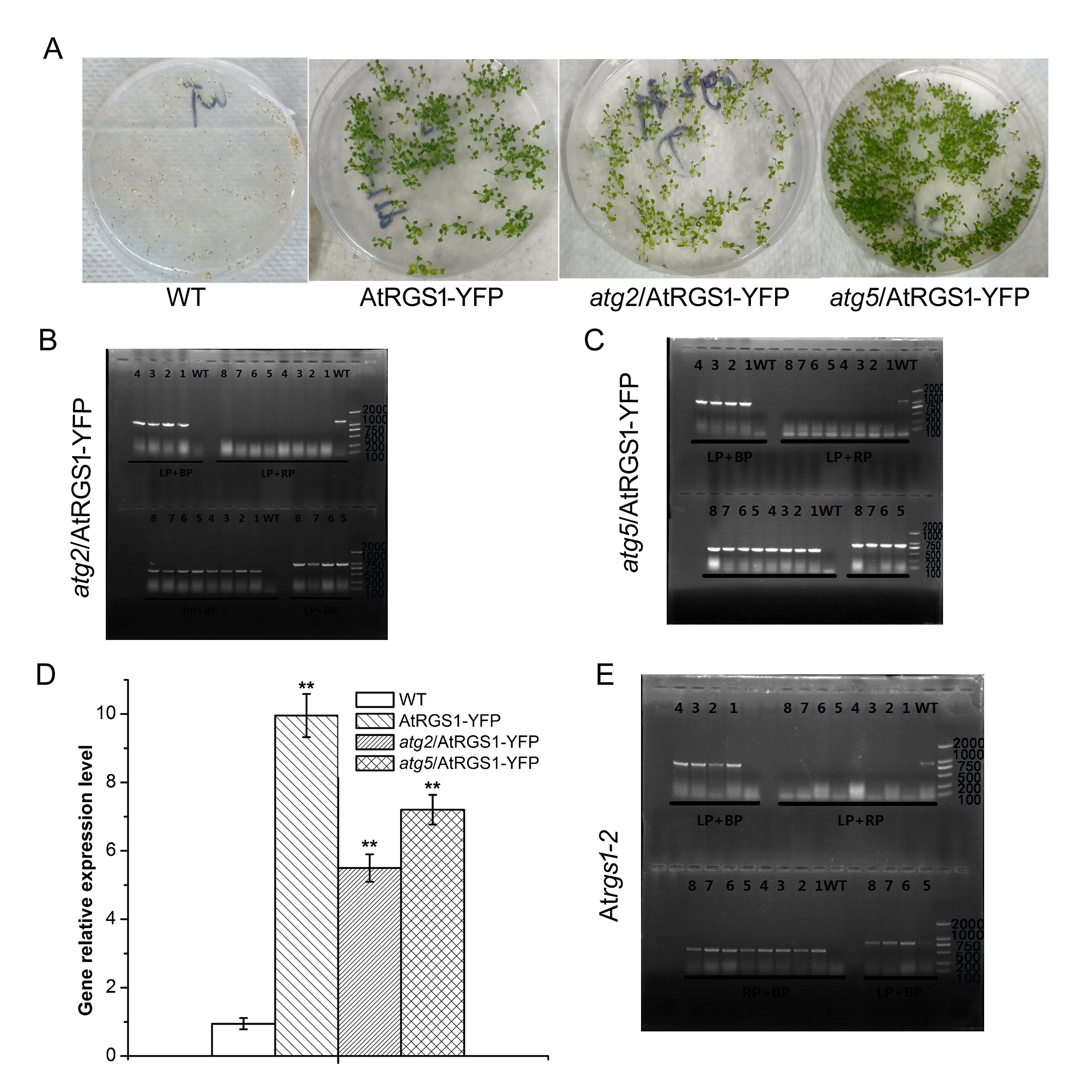

Supplement: FIGURE S1 — (A) The transgenic lines (AtRGS1-YFP, atg2/AtRGS1-YFP and atg5/AtRGS1-YFP) and WT were screened on MS plates with Basta (10 μg/mL). DNA was respectively isolated from eight seedlings (1–8) of atg2/AtRGS1-YFP (B), atg5/AtRGS1-YFP (C), and Atrgs1–2 (E). WT acts as a control. Homozygous for DNA-insertion were confirmed by PCR of genomic DNA with the primers. The products were electrophoresed on a 1% gel for 30 min, then detected by using UV illumination. (D) qRT-PCR was performed to analyze transcription level of AtRGS1 in AtRGS1-YFP, atg2/AtRGS1-YFP and atg5/AtRGS1-YFP lines grown on MS plates for 7 days. Data represent mean and SD of at least three independent experiments. The asterisk indicates a significant difference from WT (∗∗P < 0.01). [file Image_1.TIF]

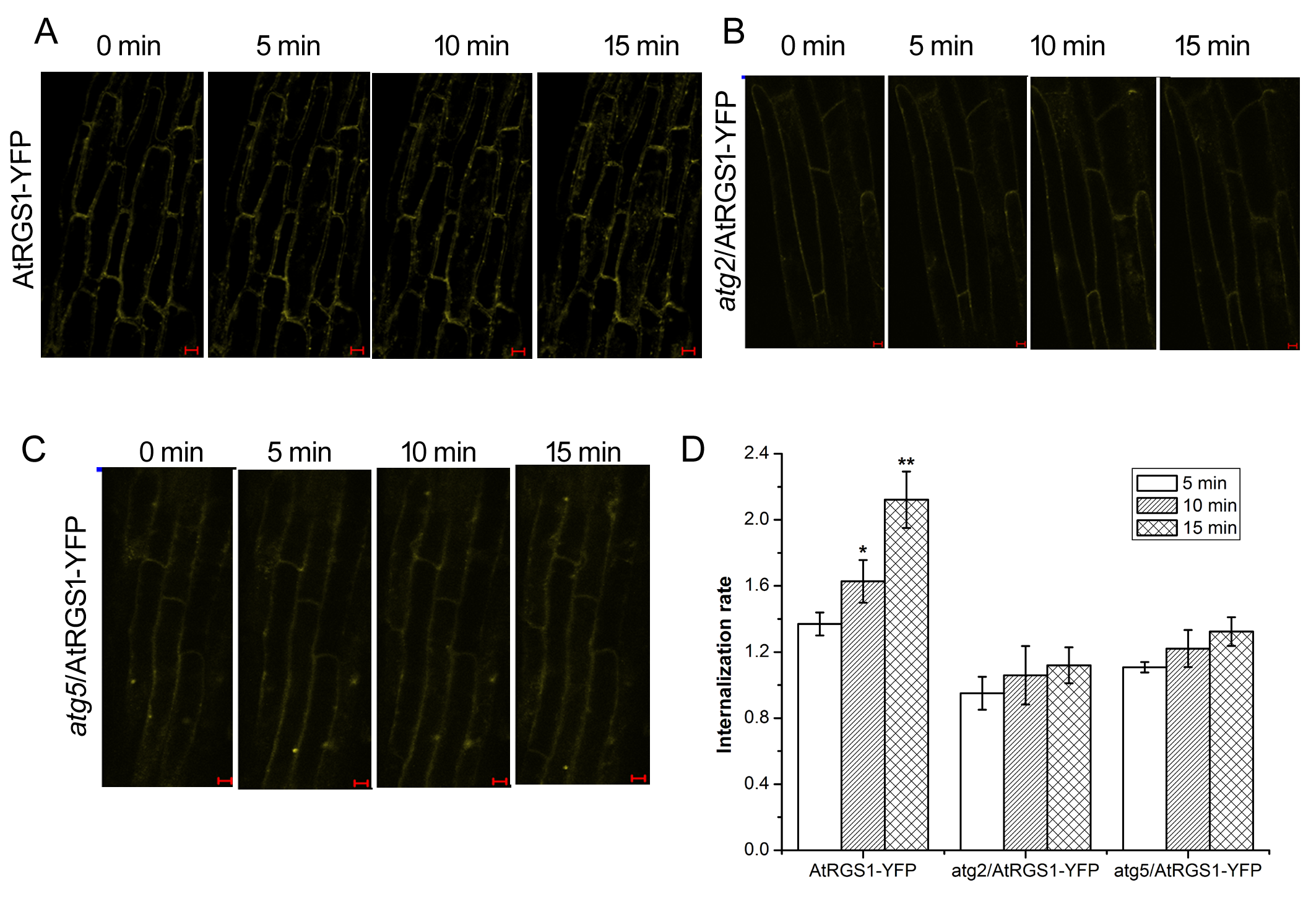

Supplement: FIGURE S2 — Movement of AtRGS1-YFP in the plant of AtRGS1-YFP (A), atg2 (B), atg5 (C). Root cells of seedlings located approximately in the elongation region were imaged using a Zeiss LSM 510 confocal laser scanning microscope (LCSM, LSM 510/ConfoCor 2, Carl-Zeiss, Jena, Germany). The normal seedlings were observation in response to D-glucose. Scale bars represent 10 μm. (D) Quantification of AtRGS1-YFP internalization rate. Internalization rate was calculated by dividing the internalization of the normal seedlings treated with D-glucose for 5, 10, or 15 min by the internalization of the normal seedlings treated with D-glucose for 0 min. Experiments were performed three independent replicates with similar results. Asterisks, significant differences from the starved seedlings treated with D-glucose for 0 min, ∗P < 0.05 or ∗∗P < 0.01. [file Image_2.TIF]
