# Supplementary material for: Endocytosis of AtRGS1 Is Regulated by the Autophagy Pathway after D-Glucose Stimulation
Source: Front Plant Sci. 2017 Jul 12;8:1229. doi: 10.3389/fpls.2017.01229 (PMC5506085; doi:10.3389/fpls.2017.01229)
Supplement: Supplementary file 3 [file Table_1.DOC]

**Supplementary Table S1 Primers used for PCR**

| **Gene name** | **AGI Number** | **Primers (5′→3′)** |
| --- | --- | --- |
| *At ATG1* | AT3G61960 | F: ATTTGAATTCACCTGCACCA |
|  |  | R: GGCAGCACTTGTTTCGTTTA |
| *AtATG2* | [AT3G19190.1](https://www.arabidopsis.org/servlets/TairObject?type=aa_sequence&id=6530308662) | F: GGATCTTCGGAGCTGCAATTA |
|  |  | R:GCAAGGACATAGAGAGCATGAA |
| *AtATG4a* | AT2G44140 | F: CAACTTCTGGGCATAAGCAA |
|  |  | R: ACGCTCCTGGAATCTCCTAA |
| *AtATG4b* | AT3G59950 | F: AAATCGATCTTCCGCAATTC |
|  |  | R: GATCGTTGAGGCTGACTTGA |
| *AtATG5* | AT3G51830.1 | F: GACCTCGTAAGGTACGGGAA |
|  |  | R: CCACTGTGTAGCGACCACTT |
| *AtATG6* | AT3G61710.1 | F: TTGCAAATTCAAAGGACCAA |
|  |  | R: TGGTCCAACTCTCTTGCTTG |
| *AtATG12a* | AT1G54210.1 | F: CGAGCTCTGTTCGGAAAGTT |
|  |  | R: CAACGAATCAGAATGAAGCTG |
| *AtRGS1* | AT3G26090 | F:CAATAGAAATGGCGAGTGGATGTGC |
|  |  | R:CGAGGAGCCTTATGAATCAAACACG |
| *Actin* | [AT3G62250](http://www.arabidopsis.org/servlets/TairObject?id=39733&type=locus) | F: GACGCTTCATCTCGTCC |
|  |  | R: GTAAACGTAGGTGAGTCCA |
| *atg5* | AT5G17290 | LP:AAAGACCACAGAACCCGAAAC |
|  |  | RP:CCAAATTGAATCTTCACCAGG |
| *atg2* | AT3G19190 | LP:GTGGGGCTCATAGCTTAGACC |
|  |  | RP:TCGAGTGATTCTGTGGTTTCC |
| *rgs1-2* | AT3G26090 | LP: TGTTGATGAAAAGCCTTAGCG |
|  |  | RP:TAGCTGCTACGCTGGAGAAAC |
|  |  | Transfer DNA_LB:ATTTTGCCGATTTCGGAAC |
